# Factors affecting mortality of hospitalized facial trauma patients in Al-Ain City, United Arab Emirates

**DOI:** 10.1371/journal.pone.0278381

**Published:** 2022-11-29

**Authors:** Mohamed A. Al-Ali, David O. Alao, Fikri M. Abu-Zidan

**Affiliations:** 1 Department of Surgery, College of Medicine and Health Sciences, United Arab Emirates University, Al-Ain, United Arab Emirates; 2 Department of Otolaryngology, Al-Ain Hospital, Al-Ain, United Arab Emirates; 3 Department of Internal Medicine, College of Medicine and Health Sciences, United Arab Emirates University, Al-Ain, United Arab Emirates; 4 The Research Office, College of Medicine and Health Sciences, United Arab Emirates University, Al-Ain, United Arab Emirates; Al Mansour University College-Baghdad-Iraq, IRAQ

## Abstract

**Background:**

Facial injuries affect one-third of severely injured patients. These injuries have devastating long-term negative impacts on quality of life. We aimed to study the epidemiology of facial injuries and factors affecting the mortality of hospitalized facial trauma patients in Al-Ain City, United Arab Emirates.

**Methodology:**

This is a retrospective analysis of prospectively collected data from Al-Ain Hospital Trauma Registry. All patients with facial injury who were hospitalized for more than 24 hours or who died after arrival at the hospital during the period from January 2014 to December 2017 were studied. Two sample data analysis was used to compare patients who died and those who survived. Significant factors were then entered into a backward logistic regression model to define factors affecting mortality.

**Results:**

408 patients having a mean age of 31.9 years were studied, 87.3% were males. The main mechanisms of injury were road traffic collisions (52.2%) and fall from height (11.3%). 289 (70.8%) patients had associated injuries which were mainly in the head and chest. The backward logistic regression model showed that the Glasgow Coma Scale (GCS) was the only factor that predicted mortality, p<0.0001 with the best cut-off point of 7.5, having a sensitivity of 0.972 and a specificity of 0.8. The ROC had an area under the curve of 0.924.

**Conclusion:**

The majority of facial injury patients in our setting are young males who were involved in road traffic collisions or falls from height. The most important factor predicting the mortality of these patients was the low GCS. Those having a GCS of 8 and more had a better chance of survival. This information is very important when counseling patients or their relatives for facial surgery.

## Introduction

Trauma is a major public health problem. One-fourth of the severely injured patients have facial injuries [1]. These injuries have devastating long-term negative impacts on quality of life and may need multiple constructive and plastic surgery with lengthy hospital stay [2–4]. They are usually associated with serious life-threatening injuries to the head and chest [5, 6]. Their mechanisms of injury include road traffic collisions (RTCs), assaults, sports injuries, falls, industrial injuries [2–4], and animal-related injuries [7]. The epidemiology of facial injuries varies worldwide including the prevalence, cause, injury pattern, severity, and clinical outcome depending on the socio-economic status and culture of the population [8]. Studies on the pattern and predictors of mortality from facial trauma are sparse. Death from facial injuries is preventable. This may occur from a compromised upper airway or bleeding from major vessels of the head and neck. Furthermore, patients with facial injuries may have associated potentially life-threatening conditions that are missed or underestimated. There are few reports studying the predicting abilities of severity scores in facial injury patients. Facial injuries are decreased in helmeted motorcyclists and bicycle riders [9, 10]. Studies varied in finding the best predictor of mortality, whether it is the injury severity score (ISS) or Glasgow Coma Scale (GCS) [11]. Lack of competence makes the assessment of facial injuries difficult both in the prehospital and hospital setting. Defining predictors of mortality may help in both triaging and priorities of transportation and management [2, 5]. Early recognition of associated injuries in patients with facial injuries is important for patient stabilization and improved clinical outcome.

This information is important to provide a basis for comparison, improve patient management including counseling, and develop injury prevention strategies. We aimed to study the epidemiology of facial injuries and factors affecting the mortality of hospitalized facial trauma patients in Al-Ain City, United Arab Emirates (UAE).

## Materials and methods

### Ethical considerations

The Human Research Ethics Committee of Al-Ain Hospital, Al-Ain, UAE gave ethical approval for this study (AAHEC-03-20-008). All admitted patients to Al-Ain Hospital or their caregivers sign a hard copy consent form on admission approving the collection of their data for clinical research. This form is part of the clinical file of the patient. The needed data for the trauma registry are then collected in a separate electronic trauma registry database as part of clinical auditing and care. Data in this registry can be used for research purposes after obtaining the ethical committee approval. The Human Research Ethics Committee of Al-Ain Hospital, Al-Ain, UAE, gave ethical approval for this study (AAHEC-03-20-008) in which anonymous data were used for the analysis.

### Study design

This is a retrospective analysis of prospectively collected data from Al-Ain Hospital Trauma Registry for a cohort of trauma patients who were followed till discharge from the hospital or death on arrival or during their stay in the hospital.

### Patients

All patients having a facial injuries who were hospitalized in Al-Ain Hospital for more than 24 hours or who died after their arrival to the hospital during the period from January 2014 to December 2017 were studied. Facial injury was defined as the presence of any injury to the mouth, eyes, nose, ears, or facial bone. Head injury was defined as the presence of any injury to the skull or brain [12].

### Setting

Al-Ain Hospital is a major university-affiliated hospital specialized in trauma and acute care. It treated approximately 80% of the hospitalized trauma patients in Al-Ain City during the study period. It is located in Al-Ain City, which has a population of 738,000 inhabitants [13].

### Studied variables

This included demography, transportation mode, mechanism of injury, anatomical location and severity of the injury, associated injuries, vital signs, Glasgow Coma Scale (GCS) on admission, Intensive care unit (ICU) admission, length of hospital stay, and clinical outcome. The severity of the injury of an anatomical region was assessed by the Abbreviated Injury Severity Score (AIS). Overall injury severity was evaluated using the Injury Severity Score (ISS) and New Injury Severity Score (NISS). Both were calculated manually, using the AIS 2008 handbook, as the sum of the squares of the highest AIS score in each of the three most severely injured body regions and the sum of squares of the highest AIS score of the three most severe injuries regardless of the body region, respectively [12]. This scoring system is internationally recognized and correlates well with different trauma outcomes such as mortality and hospital stay.

### Statistical analysis

Data were presented as mean and standard deviation (SD) for continuous data, median (range) for ordinal data, or number (%) for categorical data. Pearson’s Chi-square or Fisher’s Exact test, as appropriate, was used to compare categorical data of two independent groups and Mann–Whitney U-test for continuous or ordinal data for two independent groups. Two-sample data analysis was used to compare patients who survived and those who died. Significant factors were then entered into a backward stepwise logistic regression model. A p-value of < 0.05 was accepted as significant. Receiver operator curve (ROC) analysis was applied for significant ordinal or continuous factors to define the best point predicting mortality and its sensitivity and specificity. Statistical analyses were performed using the Statistical Package for the Social Sciences (IBM-SPSS version 26, Chicago, Il).

## Results

Four hundred eight patients were studied; 356 (87.3%) were males. The mean (SD) age was 31.9 (16.2) years; sixty-one patients (15%) were children under the age of 18. Ninety-four patients (23%) were UAE nationals. The most common mechanism of injury was road traffic collision (RTC) in 213 patients (52.2%), mainly due to car collisions (45.1%). Other etiologies were falling from height (11.3%) and falling (10.5%). The majority of patients (49.9%) were injured on the street or highway, 22.7% at home, and 15.2% at the workplace (Table 1).

**Table 1:** Two-sample data analysis comparing the demography of hospitalized facial injury patients who died and those who survived during the period of 2014–2017 at Al-Ain Hospital, Al-Ain City, United Arab Emirates.

Variable	All patients	Alive n = 397	Dead n = 11	P-value
Age (years)	31.9 (16.2)	31.8 (16.3)	37.6 (11.7)	0.14
Gender				0.99
Male	356 (87.3%)	346 (87.2%)	10 (90.9%)	
Female	52 (12.7%)	51 (12.8%)	1 (9.1%)	
Nationality				0.99
UAE nationals	94 (23%)	92 (23.2%)	2 (18.2%)	
Non-UAE	314 (77%)	305 (76.8%)	9 (81.8%)	
Mechanism of injury				0.63
Car collision	184 (45.1%)	176 (44.3%)	8 (72.7%)	
Fall from height	46 (11.3%)	44 (11.1%)	2 (18.2%)	
Fall down	43 (10.5%)	43 (10.8%)	0 (0%)	
Motorcycle collision	19 (4.7%)	19 (4.8%)	0 (0%)	
Heavy object	16 (3.9%)	16 (4%)	0 (0%)	
Bicycle collision	10 (2.5%)	10 (2.5%)	0 (0%)	
Others	90 (22.1%)	89 (22.4%)	1 (9.1%)	
Location of trauma				0.52
Street/Highway	200 (49.9%)	191(49%)	9 (81.8%)	
Home	91 (22.7%)	90 (23.1%)	1 (9.1%%)	
Work	61 (15.2%)	60 (15.4%)	1 (9.1%)	
Public area	25 (6.2%)	25 (6.4%)	0 (0%)	
Others	24 (6%)	24 (6.2%)	0 (0%)	

Data are presented as mean (SD) for continuous data and number (%) for categorical data. The numbers may not add to the total number of patients because of missing data. The valid percentages are from those available data and not the overall population

**Fig 1:**
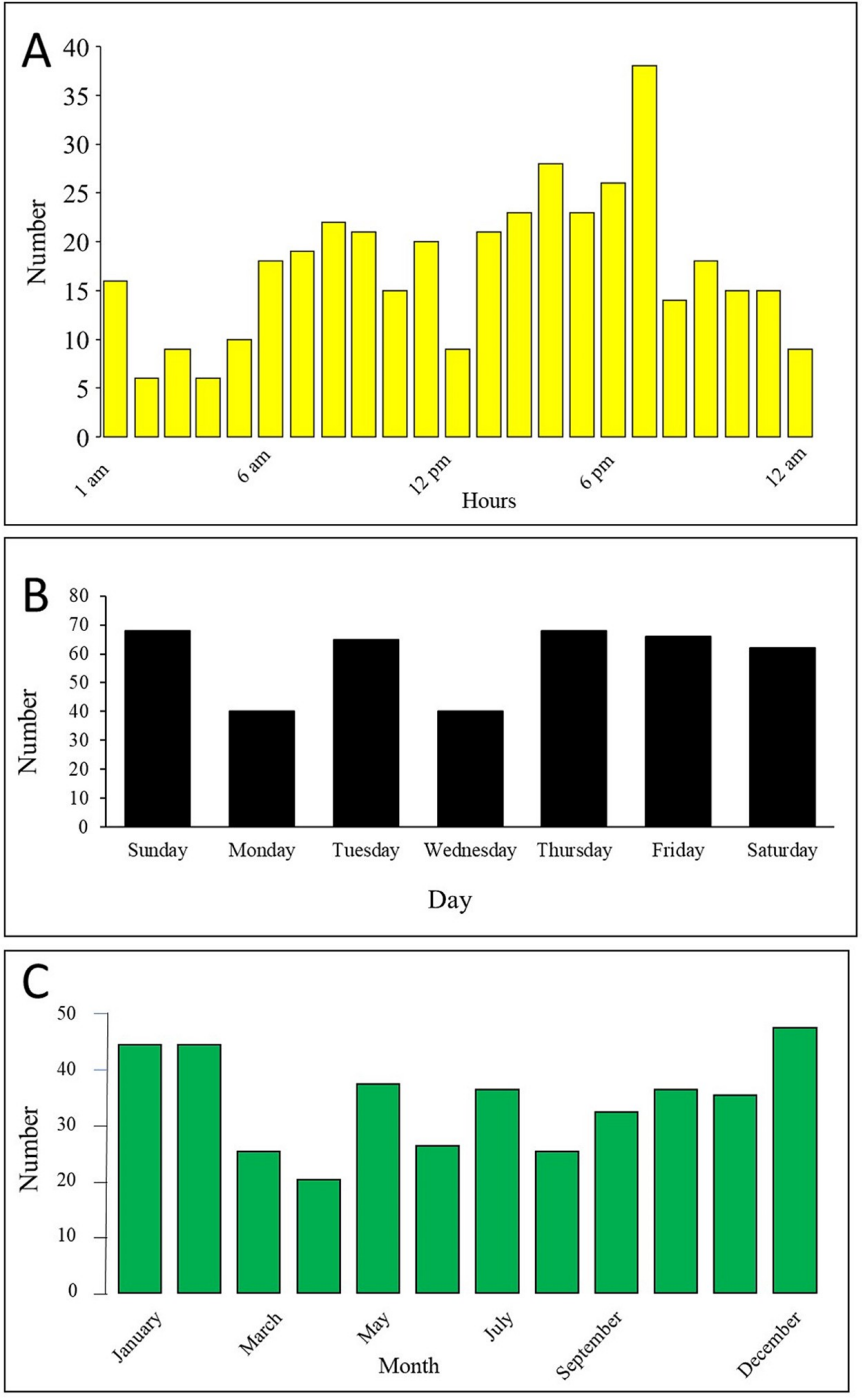
Distribution of hospitalized face trauma patients (n = 408) by time (A), day (B) and month (C), Al-Ain Hospital, Al-Ain, United Arab Emirates, during the period of 2014–2017.

The most frequent time for face injuries was at around 7 p.m. The weekly profile of facial injuries showed no difference in the incidence of injuries between the days of the week. Face injuries peaked in the winter months; December, January, and February (Fig 1).

One hundred nineteen (29.2%) patients sustained isolated face injuries, while 289 (70.8%) patients had associated injuries in other regions. The median (range) Face AIS was 1 (1–3). The most commonly injured body regions associated with face injury were the head (49.4.4%), followed by the chest (39.7%). The median (range) ISS was 5 (5–75), and the median (range) GCS was 15 (3–15). Patients stayed for a median (range) of 3 (1–95) days in the hospital. Fifty-two patients (12.7%) were admitted to the ICU. Eleven patients died (overall mortality 2.7%).

Two-sample data analysis showed that there was no statistically significant difference in the demography between those who survived and those who died including age, sex, nationality, and mechanism of injury (Table 1). Table 2 compares the severity markers between the two groups and shows that patients who died had significantly lower GCS, and significantly higher ISS, percentage of chest injury, and ICU admission (Table 2). The backward logistic regression model (Table 3) showed that the model was highly significant (p <0.0001, R squared 0.46). GCS was the only factor that predicted mortality, p<0.0001.

**Table 2:** Two-sample data analysis comparing the severity markers of hospitalized facial injury patients who died with those who survived during the period of 2014–2017 at Al-Ain Hospital, Al-Ain City, United Arab Emirates.

Variable	Alive n = 397	Dead n = 11	P-value
SBP (mmHg)	136.5 (20.6)	108.9 (61.2)	0.32
Heart rate	89.1 (18.5)	89.3 (40)	0.7
Respiratory rate	19.35 (3.2)	21.5 (10.6)	0.15
GCS	15 (3–15)	4.5 (3–15)	<0.0001
Head injury	137 (34.5%)	6 (54.5%)	0.2
Head AIS	2 (1–5)	3.5 (2–5)	0.06
Chest injury	108 (27.2%)	7 (63.6%)	0.014
Chest AIS	3 (1–4)	3 (1–4)	0.4
Spo2	98.3 (3.1)	89.6 (29.9)	0.17
ISS	5 (1–75)	18.5 (5–45)	0.001
NISS	6 (1–75)	17 (9–50)	0.001
ICU admit	2 (0.5%)	9 (81.8%)	<0.0001

SBP: Systolic blood pressure, ICU: intensive care unit, GCS: Glasgow Coma Scale, ISS: Injury Severity Score; NISS: New Injury Severity Score

**Table 3:** Backward logistic regression model showing the predicting factor of mortality in patients having facial injuries.

Variable	B	S.E.	Wald	Sig.	Exp (B)	95% CI Lower limit	95% CI Upper limit
GCS	-0.48	0.08	32.6	<0.0001	0.64	0.55	0.75
Constant	1.415	0.71	3.92	0.05	4.12		

GCS: Glasgow Coma Scale

**Fig 2:**
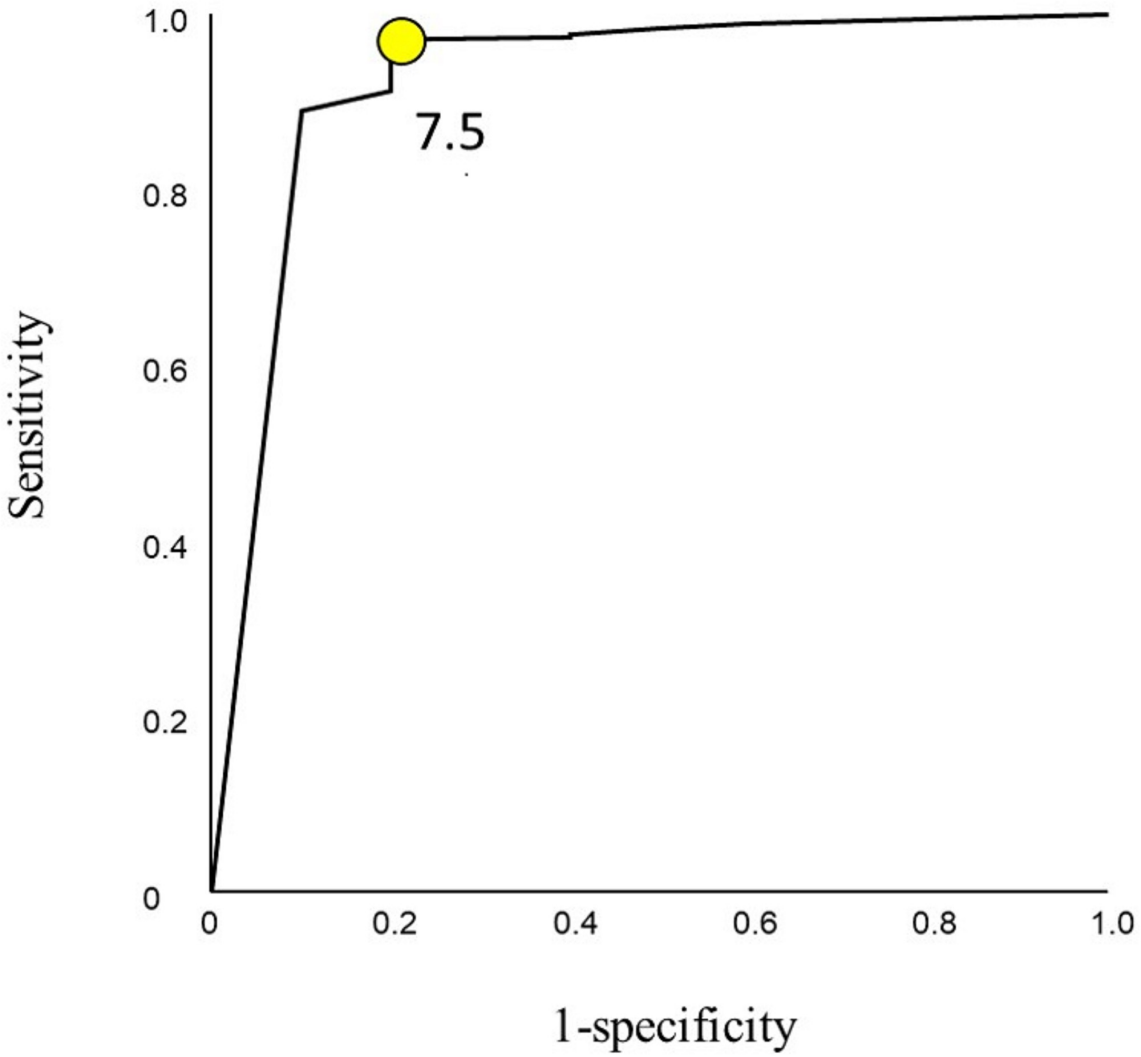
Receiver Operating Characteristic (ROC) curve for the best cut-off point of Glasgow Coma Scale (GCS) that predicts survival.

Fig 2 shows the ROC curve of GCS in predicting survival. The best cut-off point of GCS that predicted survival was 7.5, with a sensitivity of 0.972 and a specificity of 0.8. The ROC had an area under the curve of 0.924. The R squared of 0.46 indicates that the GCS can explain 46% of the variation of the model.

## Discussion

Our study has shown that more than 55% of those having facial injuries in our setting are young males who were involved in road traffic collisions or falls from height. Severe injuries that necessitated admission to the ICU with reduced GCS were highly significant in those who died. The most important factor predicting mortality of patients having facial injuries was the low GCS. Those having a GCS of 8 and more had a better chance of survival. This can be explained by the association between facial and head injuries. This information is very important when counseling patients or their relatives for facial surgery.

Similar to others, young males were the highest risk group for facial injuries in our study [1–3]. Nevertheless, the male-to-female ratio was 6.8:1, which is much higher than in other studies [14–16]. The demography of our setting is unique. The fast economic growth of the UAE required the employment of male foreign workers for construction projects. They constitute 78% of the population [9]. Similar to others, RTC is the leading cause of facial injuries [3, 17, 18]. RTC is the second cause of death in the UAE [19] which is caused by aggressive driving and poor seatbelt compliance [20, 21]. This was followed by a fall from a height, which is caused by low safety measures in the workplace [22]. In contrast to other studies [8], age had no effect on mortality in our study. This can be explained by our young population because most of expatriate workers return to their home country when they become old.

The highest number of injuries occurred around 7:00 p.m because Al-Ain City traffic is highly active during this time. Our city is spread horizontally over an area of 30 km by 25 km because it is not allowed to have more than four-story buildings in the residential areas. There are wide highways of 3-lanes having a speed limit of 80km/hour within the city. In contrast to others [23, 24], there was no increase in the incidence of facial injuries during the weekends. Furthermore, facial injuries peaked in winter. Winter in the UAE is the most pleasant season with nice weather encouraging families to have desert sports activities using quad bike riding, which has a high frequency of facial injuries [25]. In contrast, facial injuries are more common in the summer months in China and Canada [23, 24].

Associated injuries are common in patients having facial injuries [6, 5, 26]. They occurred in around 70% of our patients. There is a strong association between the presence of concomitant injuries and the trauma mechanism [5, 27, 28]. Head and chest injuries are common in our study. They occur in high-speed RTCs when not using seatbelts [29]. Traumatic brain injury (TBI) occurs in around 35% of facial trauma patients. Therefore, a high index of suspicion of TBI should be raised when evaluating patients with facial injuries [26, 30, 31]. Alvi et al. found that chest injury was the second most common associated injury and occurred in around 30% of facial trauma patients. Accordingly, trauma CT of the head and chest should be performed in patients with severe facial injuries [2]. Acute care physicians look for associations between different body regions to help them in diagnosing injuries. For example, the probability of a solid abdominal organ injury is higher when there is a lower chest injury [32]. The probability of having a bowel injury is higher when having a seatbelt sign (abdominal wall injury) and a fractured spine [33, 34]. Accordingly, the proximity of the head and chest to the face increases their probability of being injured. This simple clinical reasoning is supported by the findings of the current study.

The median length of hospital stay in our study was three days compared with 7.5 days of Mijiti *et al*. [27]. Those with pan-facial fractures and cranial injuries stay longer in the hospital [2–5]. The overall mortality in our study was 2.7% which is similar to others [5, 26]. The two-sample data analysis showed that low GCS, increased ISS, associated chest injury, and ICU admission were significantly more in those who died, similar to other studies [26, 30]. Nevertheless, the logistic regression model showed that GCS was the only significant factor predicting death, the best threshold being below 8. This highlights the importance of GCS level in predicting mortality in our study population. GCS replaced ISS, NISS, chest trauma, and admission to the ICU when put in the same logistic regression model because GCS was stronger, and because they were related variables (co-linearity effect). In other words, the severity of the head injury is related to all these four variables. Accordingly, the strongest predictor will stay in the model while the others will be removed. Similarly, Shumynskyi *et al*. found a significant association between the severity of the facial injury and brain trauma. Furthermore, they demonstrated that combinations of fractured facial bones were associated with more severe intracranial injuries, as indicated by the GCS, compared with isolated facial bone fractures [5]. This can be attributed to the proximity and direct transfer of forces from the facial skeleton to the neurocranium resulting in severe brain injury.

There is a need for more injury prevention strategies in our community targeting RTCs and work-related injuries. This includes vehicle speed monitoring, strict penalties against road traffic violations, compulsory usage of seatbelts, helmet usage by motorcyclists, and construction of cycle tracks [21, 29, 35]. Furthermore, enforcing safety in workplaces is pivotal [22]. Laborers using machines are more likely to sustain work-related facial trauma than office workers.

### Limitations

We have to acknowledge that our study has certain limitations. *First*, it is a retrospective study that can be affected by missing data. *Second*, our study did not include patients who died before arriving in the hospital or those who were treated at the Emergency Department and discharged home, which has the risk of selection bias. *Finally*, the current study stemmed from one trauma center limiting its generalizability to the whole UAE. Nevertheless, the results provide useful information to guide strategies for counseling and injury prevention.

## Conclusions

The majority of patients having facial injuries in our setting are young males who were involved in road traffic collisions or falls from height. The most important factor predicting the mortality of these patients was the low GCS. Those having GCS of 8 and more had a better chance of survival. This information is very important when counseling patients or their relatives for facial surgery.
